# An *in-silico* analysis *of OGT* gene association with diabetes mellitus

**DOI:** 10.1186/s13104-024-06744-5

**Published:** 2024-03-27

**Authors:** Abigail O. Ayodele, Brenda Udosen, Olugbenga O. Oluwagbemi, Elijah K. Oladipo, Idowu Omotuyi, Itunuoluwa Isewon, Oyekanmi Nash, Opeyemi Soremekun, Segun Fatumo

**Affiliations:** 1 H3Africa Bioinformatics Network (H3ABioNet) Node, Centre for Genomics Research and Innovation, NABDA/FMST, Abuja, Nigeria; 2 The African Computational Genomics (TACG) Research Group, MRC/UVRI, and LSHTM, Entebbe, Uganda; 3 Department of Computer Science and Information Technology, Faculty of Natural and Applied Sciences, Sol Plaatje University, 8301 Kimberley, South Africa; 4 Department of Mathematical Sciences, Stellenbosch University, 7602 Stellenbosch, South Africa; 5 Laboratory of Molecular Biology, Immunology and Bioinformatics, Department of Microbiology, Adeleke University, 232104 Ede, Nigeria; 6 Genomics Unit, Helix Biogen Institute, 210214 Ogbomoso, Nigeria; 7 Institute for Drug Research and Development, S.E. Bogoro Center, Afe Babalola University, Ado Ekiti, Nigeria; 8 Molecular Biology and Molecular Simulation Center (Mols&Sims), Ado Ekiti, Nigeria; 9 Computer and Information Sciences Department, Covenant University, Ota, Ogun State Nigeria; 10 MRC/UVRI and London School of Hygiene and Tropical Medicine London (LSHTM) Uganda Research Unit, Entebbe, Uganda

**Keywords:** Single nucleotide polymorphism (SNPs), O-linked N-acetylglucosamine transferase (*OGT*), 3 and 4

## Abstract

O-GlcNAcylation is a nutrient-sensing post-translational modification process. This cycling process involves two primary proteins: the O-linked N-acetylglucosamine transferase (*OGT*) catalysing the addition, and the glycoside hydrolase OGA (O-GlcNAcase) catalysing the removal of the O-GlCNAc moiety on nucleocytoplasmic proteins. This process is necessary for various critical cellular functions. The O-linked N-acetylglucosamine transferase (*OGT*) gene produces the *OGT* protein. Several studies have shown the overexpression of this protein to have biological implications in metabolic diseases like cancer and diabetes mellitus (DM). This study retrieved 159 SNPs with clinical significance from the SNPs database. We probed the functional effects, stability profile, and evolutionary conservation of these to determine their fit for this research. We then identified 7 SNPs (G103R, N196K, Y228H, R250C, G341V, L367F, and C845S) with predicted deleterious effects across the four tools used (PhD-SNPs, SNPs&Go, PROVEAN, and PolyPhen2). Proceeding with this, we used ROBETTA, a homology modelling tool, to model the proteins with these point mutations and carried out a structural bioinformatics method– molecular docking– using the Glide model of the Schrodinger Maestro suite. We used a previously reported inhibitor of *OGT*, OSMI-1, as the ligand for these mutated protein models. As a result, very good binding affinities and interactions were observed between this ligand and the active site residues within 4Å of *OGT*. We conclude that these mutation points may be used for further downstream analysis as drug targets for treating diabetes mellitus.

## Introduction

The human O-linked N-acetylglucosamine transferase (*OGT*) gene is ∼43 kb long. Located at the Xq13.1 genomic locus, it is alternatively spliced to generate nucleocytoplasmic (nc), mitochondrial (m), and short (s) isoforms. The varying number of tetratricopeptide repeats (TPRs) in their N-terminal domains distinguishes these isoforms. The full-length human nucleocytoplasmic *OGT* isoform (∼110 kDa) contains 13 TPRs, while mitochondrial *OGT* (∼103 kDa) and short *OGT* (∼75 kDa) contain 9 and 3 TPRs, respectively [1, 2]. The *OGT* gene encodes the *OGT* protein.

Protein O-GlcNAc transferase (*OGT*) adds the GlcNAc moiety to cytoplasmic and nuclear proteins’ threonine and serine residues. Because it is involved in cell signalling, glucose homeostasis in the liver, and regulating the clock genes’ circadian oscillation, its absence is lethal in mice [3, 4]. Torres and Hart discovered it about 30 years ago [5], and it is linked to x-linked intellectual disability and insulin resistance in muscle and adipocyte cells when mutated [6, 7]. Its contribution to glucose metabolism via the Hexosamine Biosynthesis Pathway directly links it to diabetes mellitus [8, 9].

Diabetes mellitus (DM) is a metabolic disorder that comes in two forms: T1DM and T2DM. The defective secretion of insulin causes T1DM, while T2DM is caused by a defect in insulin action [10]. Diabetes is caused by a variety of factors, including but not limited to lifestyle, genetics, and diet. Diabetes is estimated to kill 6.7 million people worldwide in 2021, with 537 million adults living with the disease, a figure that is expected to rise to 783 million by 2045 [11].

Non-synonymous single nucleotide polymorphisms (nsSNPs) are protein amino acid substitutions [12]. As a result, this study aims to identify disease-causing and deleterious SNPs within the *OGT* gene and druggable targets to discover therapeutic drugs for diabetes mellitus via this gene. To obtain an unbiased outcome, it is sensible to evaluate the detrimental prediction of various sequence-and structure-based tools, many of which have different methodologies for variant classification. The likelihood of a SNP being harmful is high if it is projected to be so by the several different predictive tools that use different methodologies. However, the performance, precision, and accuracy of the in-silico biological and clinical predictions can be improved by combining different in-silico methods or tools.

## Materials and methods

### Data retrieval for single nucleotide polymorphisms

The *OGT* variants and SNPs were retrieved from the National Centre for Biotechnology Information’s (NCBI) dbSNPs server [14]. The SNPs were chosen based on their clinical significance, as reported by ClinVar [15].

### Investigating the functional effects of coding nsSNPs

The deleterious potential of the *OGT* nsSNPs was assessed using four significant tools: Predictor of Human Deleterious Single Nucleotide Polymorphism (PhD-SNP) [12], SNPs&Go [16], PROVEAN v1.1 [17], and Polymorphism Phenotyping v2 (Polyphen) [18]. SNPs&GO is an algorithm that predicts deleterious nsSNPs based on protein functional annotation. PHD-SNP is an online tool for predicting point mutations in protein sequences and determining the impact of these mutations [19]. The program predicts how the single-point amino acid change will cause disease. PROVEAN predicts changes in a protein’s biological functions caused by single amino acid substitutions, and a score of less than − 2.5 is predicted to be harmful.

### Analysis of protein stability of predicted OGT nsSNPs

The i-Stable 2.0 server, which includes tools such as iPTREE-STAB, I-Mutant 2.0, and MUpro, was used to predict the structure-function relationship of the SNPs [20]. The i-Mutant tool calculates the Gibbs free energy for the wild-type protein and subtracts it from the mutant form to estimate the free energy changes. The predicted values of all *OGT* mutant types may alter protein stability with associated free energy. Positive DDG values indicate that the mutated proteins are highly stable, whereas negative scores indicate less stable [21].

### Analysis of the evolutionary conservation of amino acids

The Consurf program investigates the evolutionary conservation of *OGT* amino acids. It uses a Bayesian method to determine the conserved amino acids to identify the structural and functional residues in the conserved regions [22]. The prediction of the amino acids is into a variable (range between 1 and 4), intermediate (range between 5 and 6), and conserved (range between 7 and 9) based on their scores and colour indications [23].

### Protein modelling and molecular docking

Using the protein sequence retrieved from the UniProt database, we used the ROBETTA homology modelling tool to predict the 3D structure of the *OGT* apo-protein [24]. The predicted structure was viewed using the Schrodinger Maestro v11.1 workspace and validated using the Verify-3D and ERRAT programs available in the SAVES server [25]. Schrodinger-Maestro v11.1’s Protein Preparation Wizard module was used to preprocess, optimise, and minimise the crystal structure of *OGT*. While keeping the pH at 7, structural water molecules were kept to ensure protein stability, while redundant water molecules were removed to facilitate protein-ligand binding. Hydrogens were also added to fill the gaps and mediate hydrogen bridges and electrostatic forces [26]. We used the SiteMap feature of the Schrodinger Maestro software to identify potential binding pockets on the *OGT* protein [27]. The generation of receptor grids was expedient to limit ligand docking to only the identified binding pockets [28]. The grid box had dimensions of x = -32.724, y = 51.454, and z = 83.332. The PubChem database was used to retrieve the 2D structure of OSMI-1, a small molecule inhibitor of OGT [29]. The OSMI-1 was prepared and converted to its 3D geometry prior to molecular docking using the LigPrep module of Maestro v.11.1 [30].

## Results

### nsSNPs obtained from the dbSNPs database

The discovery of disease-causing nsSNPs helps develop candidate drug therapy because they are biological markers involved in disease occurrence or progression [31, 32]. The NCBI server yielded 159 nsSNPs [33]. According to ClinVar, the retrieval favoured only SNPs with clinical significance [15].

### Identification of damaging nsSNPs in OGT

We used four (4) tools to predict the potential deleteriousness of 25 nsSNPs, with at least three (3) of the four (4) tools predicting a negative effect (Table 1). PROVEAN predicted seven (7) nsSNPs to be harmful, and using the PolyPhen-2 tool, all seven (7) nsSNPs were probably harmful, with scores ranging from 0.932 to 1.000. SNPs&GO and PhD-SNP both predicted diseased SNPs. The total number of deleterious SNPs was reduced to 7 based on their detrimental effect across all four tools (Table 2).

**Table 1:** Damaging nsSNPs from OGT

S/N	rsID	AA Change/position	PROVEAN	PhD-SNPs	SNPs&GO	PolyPhen2
1	rs766646613	R627C	-4.677 Deleterious	Disease RI-2	Neutral	0.999 probably damaging
2	rs131705060	R117C	-4.194 Deleterious	Disease RI-1	Disease RI-1	0.932 probably damaging
3	rs204042438	P879L	-9.041 Deleterious	Disease RI-6	Neutral	0.942 probably damaging
4	rs204039392	P685Q	-7.872 Deleterious	Neutral	Disease RI-4	0.994 probably damaging
5	rs204042400	R867C	-5.399 Deleterious	Disease RI- 1	Neutral	0.994 probably damaging
6	rs204034593	A380V	-3.790 Deleterious	Neutral	Disease RI-4	0.938 probably damaging
7	rs766646613	R627C	-4.677 Deleterious	Disease RI-2	Neutral	0.999 probably damaging
8	rs2040347448	M401T	-4.727 Deleterious	Disease RI-2	Neutral	0.998 probably damaging
9	rs2040347668	C417Y	-8.596 Deleterious	Neutral	Disease RI-1	0.989 probably damaging
10	rs2040350890	D481G	-4.340 Deleterious	Disease 1	Disease RI-2	benign
11	rs2040368778	H611N	-5.952 Deleterious	Disease 2	Neutral	0.55 probably damaging
12	rs2040387073	P657L	-9.335 Deleterious	Neutral	Disease RI-6	0.924 probably damaging
13	rs2040191136	Y112S	-7.489 Deleterious	Neutral	Disease RI-7	0.973 probably damaging
14	rs2040329106	Y228H	-2.680 Deleterious	Disease 5	Disease RI-0	0.997 probably damaging
15	rs2040334939	R250C	-6.093 Deleterious	Disease 3	Disease RI-3	1.000 probably damaging
16	rs2040341169	G341V	-7.294 Deleterious	Disease RI-3	Disease RI-3	0.991 probably damaging
17	rs2040345810	L367F	-3.717 Deleterious	Disease 1	Disease RI-1	0.999 probably damaging
18	rs2040190682	R102G	-4.116 Deleterious	Neutral	Disease RI-1	0.930 PROBABLY DAMAGING
19	rs2040334968	R250L	-5.405 Deleterious	Disease 2	Disease RI-0	1.000 probably damaging
20	rs772525369	R899C	-6.682 Deleterious	Disease RI-0	Disease RI-2	1.000 probably damaging
21	rs1114167891	R284P	-4.060 Deleterious	Disease 6	Disease RI-6	0.951 probably damaging
22	rs1556046834	G103R	-5.717 Deleterious	Disease 5	Disease RI-6	0.993 probably damaging
23	rs1602152230	N648Y	-7.605 Deleterious	Disease 6	Disease RI-0	0.998 probably damaging
24	rs2040405196	C845S	-7.654 Deleterious	Disease 3	Disease RI-3	0.930 probably damaging
25	rs200109331	N196K	-4.599 Deleterious	Disease 5	Disease RI-5	1.000 probably damaging

**Table 2:** Predicted deleterious nsSNPs across the four tools

S/N	rs ID	AA Change/Position	PROVEAN	PhD-SNPs	SNPs&GO	PolyPhen2
1	rs2040329106	Y228H	-2.680 Deleterious	Disease 5	Disease RI-0	0.997 PROBABLY DAMAGING
2	rs2040334939	R250C	-6.093 Deleterious	Disease 3	Disease RI-3	1.000 PROBABLY DAMAGING
3	rs2040341169	G341V	-7.294 Deleterious	Disease RI-3	Disease RI-3	0.991 PROBABLY DAMAGING
4	rs2040345810	L367F	-3.717 Deleterious	Disease 1	Disease RI-1	0.999 PROBABLY DAMAGING
5	rs2040405196	C845S	-7.654 Deleterious	Disease 3	Disease RI-3	0.930 probably damaging
6	rs1556046834	G103R	-5.717 Deleterious	Disease 5	Disease RI-6	0.993 PROBABLY DAMAGING
7	rs200109331	N196K	-4.599 Deleterious	Disease 5	Disease RI-5	1.000 PROBABLY DAMAGING

### Protein stability profile prediction for nsSNPs in OGT

The iStable 2.0 tool predicted protein stability [34]. All seven highly deleterious SNPs were also predicted to reduce *OGT* protein stability. The results of MUpro SVM, MUpro MM, I-Mutant 2.0, and iPTREE-STAB are shown in Table 3.

**Table 3:** nsSNPs stability profiling

S/N	SNPs	AA Change	I-Mutant2.0 SEQ	MUpro_SVM	MUpro_NN	iPTREE-STAB
1	rs2040329106	Y228H	Decrease	Decrease	Decrease	Decrease
2	rs2040334939	R250C	Decrease	Increase	Increase	Decrease
3	rs2040341169	G341V	Decrease	Decrease	Increase	Decrease
4	rs2040345810	L367F	Decrease	Decrease	Decrease	Decrease
5	rs2040405196	C845S	Decrease	Decrease	Decrease	Decrease
6	rs1556046834	G103R	Decrease	Increase	Increase	Decrease
7	rs200109331	N196K	Decrease	Decrease	Decrease	Decrease

### Conservation prediction of damaging nsSNPs in OGT

Consurf predicted that Y228H, C845S, and L367F would be buried and conserved, whereas G103R, N196K, R250C, and G341V would be exposed and conserved (Table 4).

**Table 4:** ConSurf result output

S/N	Amino acid change	Pos	Seq	Score	Colour	Confidence interval	Confidence interval colours	B/e	Function	Msa data	Residue variety
1	G103R	103	G	-0.148	6	-0.417, 0.041	7,5	e		122/150	G, N, A, V
2	N196K	196	N	-0.805	9	-0.861, -0.777	9,9	e	f	127/150	N, Y, S
3	Y228H	228	Y	-0.218	6	-0.417, -0.080	7,5	b		126/150	Y, L, H, F
4	R250C	250	R	-0.104	6	-0.350, 0.041	7,5	e		143/150	K, R, E, S, T, N, H, Q, A
5	C845S	845	C	-0.077	9	-0.272, 0.041	9,9	b	S	147/150	Q, H, Y, T, E, S, K, R
6	G341V	341	G	-0.703	9	-0.799, -0.660	9,8	e	f	147/150	S, G, C, N
7	L367F	367	L	-0.78	9	-0.849, -0.752	9,9	b	S	146/150	I, Y, L

### OGT structural characterisation of wild and mutant types in comparison

**Fig. 1:**
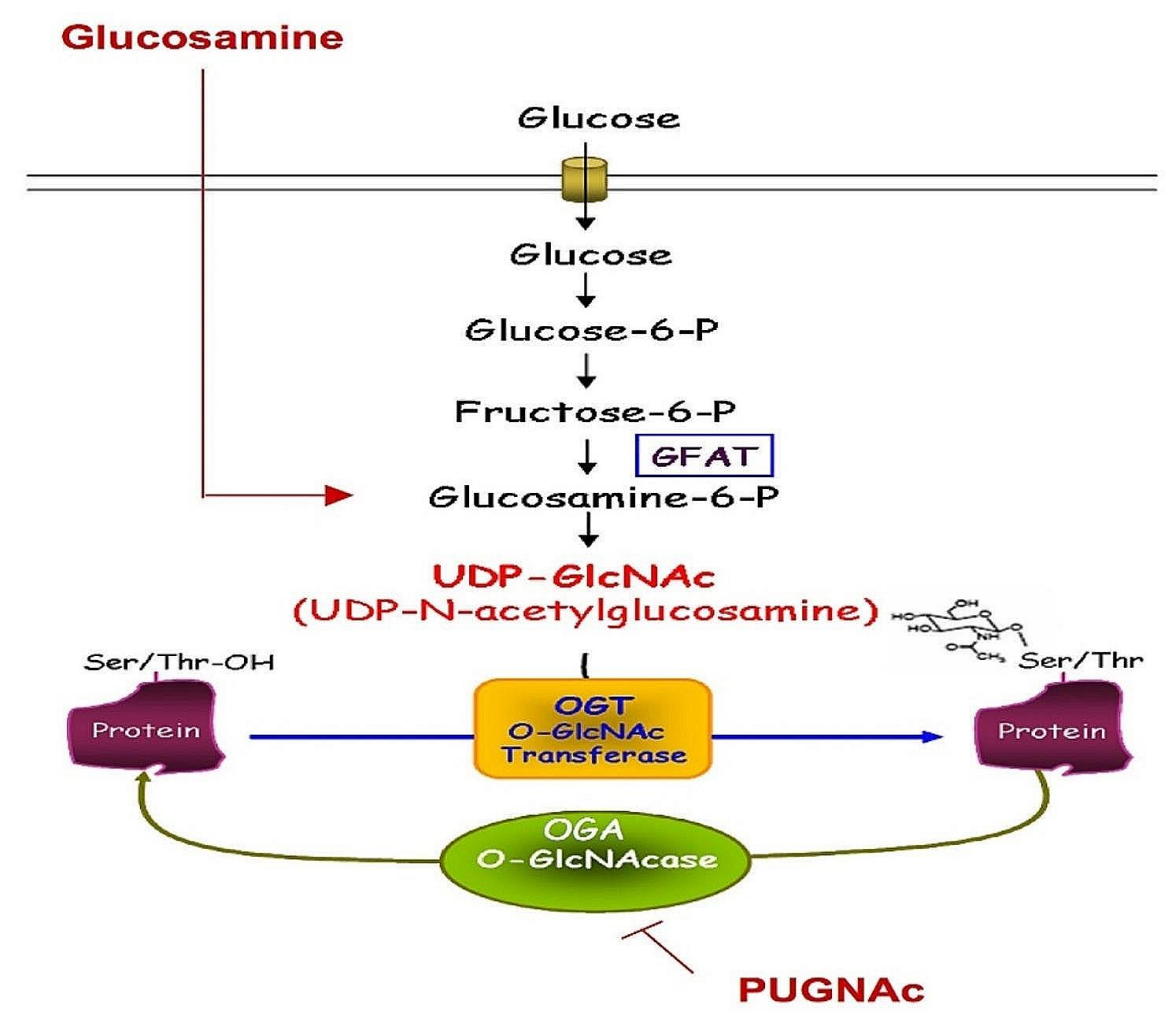
The Hexosamine Biosynthesis pathway promotes protein O-GlcNAcylation by supplying the O-GlcNAc moiety for addition and removal on nuclear and cytoplasmic proteins [13]

**Fig. 2:**
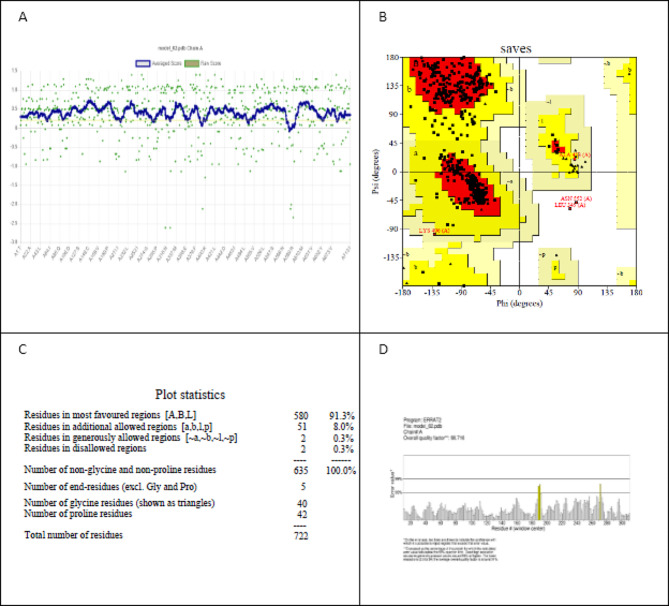
**A** Verify the 3D plot for the modelled protein, **B** Ramachandran plot showing the majority of the modelled protein’s residues in the favoured region, **C** The Ramachandran plot statistics provide values for the residues, **D** the ERRAT overall quality factor is 98.716

ERRAT and Verify-3D were used to validate the protein structure (Fig. 1). According to the Verify-3D results, 94.39% of the residues have an average 3D-ID score of 0.2. (Fig. 2a). The Ramachandran plot, which is available in PROCHECK, was used to assess the quality of the 3D protein structure (Fig. 2b). According to the plot, 91.3%, 8.0%, 0.3%, and 0.3% of the residues are in the favoured, allowed, generously allowed, and disallowed regions, respectively (Fig. 2c). This confirms the protein structure’s high quality. ERRAT also demonstrated an overall quality factor of 98.7161 (Fig. 2d), implying that the results obtained from the tools, as mentioned earlier, indicated that our modelled protein is of high quality and can be used for further investigation.

### OGT Mutant type as a potential drug target

The Glide module of the Schrödinger Maestro Suite was used to investigate the protein-ligand binding affinity of OSMI-1 and the OGT protein. OSMI-1 interacted well with the active site residues of *OGT*, and the docking scores for each interaction are shown in Table 5. These predictions can be validated using additional downstream analysis.

**Table 5:** Molecular docking results of mutant type OGT against OSMI-1

S/N	Amino acid change	Docking scores	Interacting residues
1	G103R	-4.646	GLU649, LYS534, ARG338, and ASN621
2	N196K	-5.183	LYS644, GLY645, and ASN648
3	Y228H	-5.069	LYS534, ASN621, ALA646, and TYR642
4	R250C	-4.775	HIS508, LYS852, THR932, PHE878, LYS908, HIS568, and LYS644
5	C845S	-4.571	GLU649
6	G341V	-5.145	ASN567, SER594, and LYS644
7	L367F	-5.563	GLU649, ALA646, TYR642, and LYS534

## Discussion

*OGT* gene has emerged as the candidate gene associated with diabetes mellitus [35]. However, the relationship is complex and requires consideration of various factors. Several important functional regulatory factors, including SNPs, may significantly impact disease metabolism. Utilising publicly available data, we discovered seven deleterious SNPs associated with the *OGT* gene. Additionally, we examined the functional consequences of these SNPs, conservation analysis, protein-protein interaction network studies, and protein stability. The *OGT* gene is crucial in diverse cellular processes, including metabolism, insulin signalling, and stress response. Due to their potential effects on protein structure and function and, eventually, cellular processes involved in glucose metabolism and insulin signalling, deleterious single nucleotide polymorphisms (SNPs) in the OGT gene may have a major impact on diabetes. Our study shows that only the mutation points in G103R, Y228H, R250C, C845S, G341V, N196K, and L367F were found to be harmful across all four tools used, out of the 25 deleterious nsSNPs identified.

Furthermore, we characterised the identified SNPs based on their stability. Protein stability is essential for maintaining these functions. Meanwhile, unstable proteins are more susceptible to degradation by cellular machinery, reducing *OGT* levels and activity. A protein’s function is determined by changes in its conformational structure, which is influenced by changes in protein stability [36]. Our study shows that the protein stability of the *OGT* gene is impacted by the identified nsSNPs, which may negatively impact the protein’s structure and function. Decreased protein stability can alter how proteins fold, leading to abnormal protein aggregation or increased degradation [37].

Based on similarity and homology data, Consurf calculates the evolutionary profile of proteins and the effects of amino acid substitutions [23]. The evolutionary profiling of the *OGT* SNPs predicted all seven to be located in the conserved region. Y228H, G103R, N196K, R250C, G341V, L367F, and C845S amino acids substitute for rs2040329106, rs1556046834, rs200109331, rs2040334939, rs2040341169, rs2040345810 and rs2040405196 (Table 4). SNPs in these areas can significantly alter protein structure and function, potentially leading to disease or altered phenotype [38]. It emphasises its potential significance for understanding disease mechanisms and developing novel therapeutic strategies. Conserved regions often encode crucial parts of proteins, like active sites or binding pockets. Because the nsSNPs were found in a conserved region, a change in the amino acid sequence in those regions will affect the structural and functional profile of the *OGT* protein.

Our molecular docking analysis indicated that all docking scores vary between the mutants, ranging from − 4.546 to -5.563, suggesting differential binding strengths. The higher the score, the stronger the predicted binding affinity (Table 5) [39]. Overall, our docking results provide valuable insight into the potential impact of *OGT* mutations on OSMI-1 binding. Further experimental validation and functional analysis are crucial for conclusively understanding their effects on *OGT* activity and biological significance.

The current study’s strength lies in using various algorithms to obtain precise prediction results for the identified nsSNPs. These could be used as druggable reference points to discover drugs to treat diabetes mellitus. There is a need to investigate more reliable in-vitro and in-vivo investigations to corroborate these results. A significant limitation of this work, like other in-silico studies, is that all of the processes employed to predict the impact of the SNPs are computer-based.

## Conclusions

The *OGT* protein has been linked to the progression of diabetes mellitus because it catalyses the addition of the o-GlcNAc sugar moiety on nucleocytoplasmic proteins, a substrate of the hexosamine biosynthesis pathway, increasing the amount of intracellular glucose content. In this study, 159 *OGT* nsSNPs in coding regions were chosen, and structural analysis of the seven nsSNPs predicted a negative impact on protein function and stability. The findings indicated that nsSNPs could be used in drug development for diabetes mellitus.

## Data Availability

1. PolyPhen2; http://genetics.bwh.harvard.edu/pph2/ 2. SNPs&Go; https://snps-and-go.biocomp.unibo.it/snps-and-go/ 3. PhD-SNP; https://snps.biofold.org/phd-snp/phd-snp.html 4. PROVEAN; https://bio.tools/provean 5. SNPs database; https://www.ncbi.nlm.nih.gov/snp/ 6. Consurf; https://consurf.tau.ac.il/consurf_index.php 7. ROBETTA; https://robetta.bakerlab.org/ 8. ClinVar; https://www.ncbi.nlm.nih.gov/clinvar/ 9. ERRAT; https://www.doe-mbi.ucla.edu/errat/ 10. Verify3D; https://www.doe-mbi.ucla.edu/verify3d/ 11. SAVES; https://saves.mbi.ucla.edu/
